# Elements of Neuroanthropology

**DOI:** 10.3389/fpsyg.2021.509611

**Published:** 2021-10-12

**Authors:** Daniel H. Lende, Breanne I. Casper, Kaleigh B. Hoyt, Gino L. Collura

**Affiliations:** Department of Anthropology, University of South Florida, Tampa, FL, United States

**Keywords:** neuroanthropology, culture, neuroscience, veterans, raptors, cue reactivity, addiction

## Abstract

Neuroanthropology is the integration of neuroscience into anthropology and aims to understand “brains in the wild.” This interdisciplinary field examines patterns of human variation in field settings and provides empirical research that complements work done in clinical and laboratory settings. Neuroanthropology often uses ethnography in combination with theories and methods from cognitive science as a way to capture how culture, mind, and brain interact. This article describes nine elements that outline how to do neuroanthropology research: (1) integrating biology and culture through neuroscience and biocultural anthropology; (2) extending focus of anthropology on what people say and do to include what people process; (3) sizing culture appropriately, from broad patterns of culture to culture in small-scale settings; (4) understanding patterns of cultural variation, in particular how culture produces patterns of shared variation; (5) considering individuals in interaction with culture, with levels of analysis that can go from biology to social structures; (6) focusing on interactive elements that bring together biological and cultural processes; (7) conceptual triangulation, which draws on anthropology, psychology, and neuroscience in conjunction with field, clinic, and laboratory; (8) critical complementarity as a way to integrate the strengths of critical scholarship with interdisciplinary work; and (9) using methodological triangulation as a way to advance interdisciplinary research. These elements are illustrated through three case studies: research on US combat veterans and how they use Brazilian Jiu Jitsu as a way to manage the transition to becoming civilians, work on human-raptor interactions to understand how and why these interactions can prove beneficial for human handlers, and adapting cue reactivity research on addiction to a field-based approach to understand how people interact with cues in naturalistic settings.

## Introduction

In researching how our nervous system functions in both common and varied ways across settings, neuroanthropology has drawn on the strengths of anthropology—a holistic approach that fosters interdisciplinarity, the theory of culture, and field-based research. Neuroanthropology has also embraced the attention to the process that comes with biology and cognitive science as well as increasing understanding of neuroplasticity of neuroscience. Together, these have fostered examining “brains in the wild” (Lende and Downey, 2012a,b).

Related fields, such as cultural neuroscience and critical neuroscience, have also drawn on shifting views of the brain, while social science research has increasingly focused on how the explosion in neuroscience matters for society (Chiao and Ambady, 2007; Rose and Abi-Rached, 2013; Fitzgerald and Callard, 2015; Choudhury and Slaby, 2016; Kitayama et al., 2019). Neuroanthropology can contribute to these exciting interdisciplinary developments by offering ways to incorporate the empirical and conceptual developments made by anthropologists. By studying human variation in context, neuroanthropology helps to bridge the gap between the laboratory studies favored by neuroscience and field-based anthropology focused on sociocultural phenomena.

In this paper, we review three domains that prove important for neuroanthropological research: (1) how holism and biocultural approaches foster interdisciplinary research that draws on neuroscience and on field research (Hruschka et al., 2005; Quinn, 2018), (2) ways to incorporate culture at varying levels into a project, and (3) how different forms of triangulation offer ways to integrate different ideas and methods into research (Patton, 1999; Maxwell, 2004; Fusch et al., 2018). After covering these key elements, we provide lessons learned from three different research projects—work with veterans, human-raptor interactions, and how people react to cues for drugs—for neuroanthropology.

## Holism

### Combining Biology and Culture

Neuroanthropology starts with a reciprocal understanding of how humans work: We create culture, and culture creates us. For neuroanthropology, culture is interactive, a dynamic set of processes both outside and under the skin. This view means that enculturation—or how culture comes to be embodied in a specific person—works *via* biocultural transformations that happen over time and in particular settings (Hruschka et al., 2005; Worthman, 2009; Lende and Downey, 2012a).

Researchers who are less familiar with biological processes should recognize that the specifics of neural processing are not intuitively obvious—often times, they correspond neither to our mechanical understandings of how the world works, nor our representationalist view that there is a perceiving subject somewhere inside the brain (Lende and Downey, 2012a). For example, the popular press has covered Marilyn Monroe or Jennifer Aniston neurons, conveying the idea that specific neurons correspond to specific faces (or, more broadly, this part does that function; Abbott, 2010; Goldhill, 2016). However, research on facial recognition remains a work in progress and shows that both higher-level and lower-level kinds of processing matter for recognizing a face. For example, the fusiform face area in the dorsal visual cortex does higher-order face processing of basic visual perceptions, helping us to recognize familiar faces (Kanwisher and Yovel, 2006) even when the perceiving subject is blind from birth (Murty et al., 2020). At the same time, recent primate research also has made clear that the brain makes sense of faces by breaking faces down into separate componential processes, attending to relatively invariant sensory features that can then be used to create a composite (or constructed) face (Chang and Tsao, 2017). Thus, the specifics of how the biology actually gets a particular function done matter for doing good neuroanthropology and for linking biology to cultural science.

From the culture side, researchers less familiar with anthropology should recognize that their common-sense categories of how we perceive and act and experience the world represent western ethnopsychologies. Ethnopsychologies divvy up the world in particular ways, and that divvying up is not necessarily something natural or preexisting inside the world. Rather, it comes from culture. For example, westerners rely on a model of the five senses, each as its own separate domain from the other, which together comprise how people perceive the world. However, other cultures operate from different assumptions. For example, in Ghana, the Anlo-Ewe-speaking people view balance as a main sense and central to defining what it means to be a person (Guerts, 2003). Moreover, the Anlo-Ewe approach senses not as perception but as feeling, something that is intuited and felt within the body; in contrast, western psychology generally views perception happening at the interface of the mind and physical body. Thus, the Anlo-Ewe view of sensory perception/feeling is distinct from the mind-body dichotomy commonly used in the west.

To navigate between both a nonintuitive biology and a non-intuitive culture, neuroanthropology has drawn on neuroscience that can connect to learning, human development, and culture (Wexler, 2011; Lende and Downey, 2012a; Sherwood and Gómez-Robles, 2017). At the biological level, neural reuse provides one approach that helps to understand how the brain can repurpose existing elements as a way to solve novel tasks and support culture (Anderson, 2010). *Via* neuroplasticity acting from cellular to circuit levels, built-in functions can be adapted to new demands, and new functions can emerge from combining existing neural components with learning and environmental inputs. Culture excels at demanding exactly this sort of learning and by structuring inputs in ways that push both human (and sometimes nonhuman) brains to do novel things, such as using symbols (Matsuzawa, 2009; Whiten, 2017), reading (Dehaene-Lambertz et al., 2018), and tool use (Stout and Hecht, 2017).

Neuroconstructivism provides an approach that can connect neural function and reuse to development in specific contexts (Bates et al., 1996; Westermann et al., 2007). Neuroconstructivism focuses on the experience-dependent development of neural structures, where experience can play a fundamental role in shaping functionality and the emergence of both competent function and neuropathology (Campos et al., 2019). In turn, neural reuse and neuroconstructivism can work with embodied, enactive, and extended mind approaches. This research connects neural and cognitive processing to local contexts (Clark and Chalmers, 1998; Clark, 2008; Wilson and Golonka, 2013). As Wilson and Golonka (2013) argue, these approaches need to look at the task to be solved from the point of view of the agent, and then examine the resources available to solve that task. These resources are not just internal—brain, body, other people, symbols, and tools can all help.

Emotion can provide an example of this integrative approach. Work by Barrett and colleagues approaches emotions as constructed rather than reducible to universal patterns (Barrett, 2017; Gendron et al., 2018). In their approach, emotions still involve bodily states and brain processing but also rely on contextual cues and learned interpretations. Thus, they can vary cross-culturally rather than having discrete and distinguishable signatures in brain signals or bodily states. In this type of approach, emotions can be similar cross-culturally not just because of an innate biology but because of commonalities in our bodies and faces, how our brains process both general states (from hunger and illness to anger and anxiety) and specific emotions (say, *schadenfreude*), and similarities in social situations and recurring cues that can guide specific interpretations (Crivelli et al., 2016; Barrett et al., 2019; Srinivasan and Martinez, 2021). It is the recurring combinations, from inside to outside, that can lead to commonalities across cultures.

## Say-Do-Process

Alongside theoretical considerations of how to bring together neuroscience and anthropology, neuroanthropology builds its approach to research problems, using insights from the field. As Roepstorff and Frith (2012) argue, anthropology is well-equipped to handle competing discourses and models while also providing a focus on specific examples in concrete settings. With field research, anthropology has stressed the importance of paying attention to what people say and what people do. They are not the same thing; people might say one thing and do another. Moreover, what they say about what they do and why, does not necessarily match up with what they actually do.

Here, we add a third element—what people process. Process brings in emphasis of cognitive science on understanding mechanisms, functions, and mediating factors. Psychology has long recognized the distinction between what people say and what people process (Nisbett and Wilson, 1977). In contrast, field-based research in anthropology has often focused on higher-level sociocultural phenomena. Through a process, neuroanthropology draws on biology, cognition, and development to better understand human variation and outcomes in real-world settings. Process brings attention to how things happen, not just to what people say and do.

In research, Say corresponds best to language, symbols, metaphors, and meaning that shape how we think and speak (Hutchins, 1995; Tedlock and Mannheim, 1995; Zarger, 2010). For neuroanthropological research, getting at Say can happen through watching people speak together and doing informal interviews in field settings, as well as *via* interviews, questionnaires, and other forms of verbal response. Listening to how people interpret what happens and why, as well as developing their views on things in their own words, forms an important part of capturing Say in field research.

Do corresponds best to practices and how people interact in specific contexts. These often form the core of ethnographic research, particularly participant observation where researchers can interact informally with local people and observe firsthand how life plays out in real time. For neuroanthropology, what people do can connect senses and movement and action on the neuroscience side, and the skills and practices and doing on the anthropology side (Worthman, 2009; Downey, 2010). Neuroscience here provides a wealth of work on senses, movement, balance, and more that can effectively work with the emphasis that anthropology provides on how people behave, whether following one individual or looking at coordinated behavior in specific settings (Clark, 2008; Han, 2015; Chiao et al., 2016; Kitayama et al., 2019).

It is important, nonetheless, to view what people do as happening in public and between people. That means that culture and interactions are available for observation. Provided researchers frame questions right, questionnaires can also get at what people do by asking about specific experiences and behaviors, and getting informants to report on behaviors done within specific contexts (e.g., home, work, and other types of sociocultural contexts). Finding specific moments where things happen that crystallize insights is the mainstay of ethnographic research and equally important to field-based neuroanthropology.

What people do and what people say do not provide automatic access to how and what people process. Roepstorff and Frith (2012) argue that anthropological approaches need to pay attention to mechanism (or process) to effectively bridge laboratory and field. To understand process, ethnographic research combined with biocultural approaches can help. Understanding how development, plasticity, neural reuse, algorithmic processing and the like impact any particular problem will help to capture how people process (Anderson, 2010; Worthman, 2010; Wilson and Golonka, 2013). These processes often happen outside of conscious awareness and cultural modes of understanding but play a major role in shaping human variation. Observations on process have been proved effective in getting at process in field settings (Worthman, 2009; Xygalatas et al., 2013; McGraw and Krátký, 2017). Alongside observations, researchers can encourage informants to do thick description of what they experience, what happens as they do something, and what the context is like.

## Culture

### Sizing Culture Appropriately

After establishing a holistic approach, it is important to consider how “culture” is sized in a particular study, and how that fits any proposed interactions or effects that culture might have (see Table 1). Cultural neuroscience has generally taken a broad approach to culture, approaching “culture” as a regional or national phenomenon and linking it to changes in psychological or neural states and functions (Chiao and Ambady, 2007; Han, 2015). This research has yielded important insights. For example, societies that stress interdependence over independence show differing patterns of neural activation in self-other tasks; interdependent cultures tend to show greater activation in circuits that are generally considered “self” oriented in western contexts (e.g., greater activation in Germans than Chinese in the medial prefrontal cortex; Korn et al., 2014).

**Table 1:** Sizing culture appropriately.

**Level of granularity**	**Symbolic/ interpretive**	**Societal/ political economy**	**Ecological/ environmental**
Macro	Symbols	Political economic structures	Local ecology
Meso	Rituals	Social institutions	Food and making a living
Micro	Interpretations	Households	Interspecies interactions
Mental	Subjective meanings and models	Individual preferences and tastes	Local environmental knowledge and practices

*At a practical level, sizing culture needs to work in an iterative fashion from the broadest considerations down to the most micro to figure out what is an appropriate level of analysis for any particular project. Often the broadest orientations—symbolic, political economic, ecological—are not enough to understand variation within a particular society. Once researchers have chosen an appropriate overall approach, they will need to work down to the level of granularity that most impacts their specific research problem. The level of granularity in cultural analysis is presented on left, from large-scale considerations (the bird's eye view) down to how individuals relate to culture. On the top level, in italics, are prominent approaches to assessing culture in its broadest sense; these different orientations all represent decades-old research paradigms. The more fine-grained labels (e.g., social institutions, households, and tastes) are placemarkers to indicate the level of granularity, a label to exemplify a type of variation. Other possible research foci are possible (e.g., Mosse's, 2004 research on non-profit organizations, internal NGO organization, and cognitive contradictions in goals vs. practice). Researchers should consider which type of approach to culture best fits their research problem (Table 2), and from there, how many levels of analysis (e.g., degrees of granularity) are feasible to include in the research. Often times researchers might consider broad patterns of meaning making (language, nationality, and history) but focus in on smaller-scale aspects of culture (prominent interpretations that relate to sensory processing, and experience-near descriptions of sensory details). Interpretive, political economic, and ecological approaches are not the only ways to “size culture.” Research on globalization and on the materiality of cultural life are other prominent ways to analyze the patterning of variation; however, these approaches generally engage with one or more of these macro views on human culture*.

However, two potential problems can arise. First, such a broad generalization (east vs. west) can hide significant variation, whether within a particular group, community, or region (Lende and Downey, 2012a; Seligman et al., 2016). Second, by using a generic level of culture, broad differences are likely to be distal compared to more immediate influences on psychological and neural function. For example, context deeply shapes human behavior but is often not assessed in large-scale generalizations (Moore, 2004; Medin et al., 2010; Roepstorff et al., 2010; Northoff, 2013). Thus, large-scale sizing of culture brings advantages, from ease of research and ability to work across labs in multiple countries, and disadvantages, such as overgeneralization across groups and the underpowering of the specific effects of culture.

Neuroanthropology has generally focused on more proximate approaches, sizing culture at a more local level. Research of Lende (2005) on drug use and dopamine function framed culture at the level of shared understandings of addiction in urban areas in Colombia. An even more local approach came from the work of Downey on capoeira and proprioception focused on specific training regimes and skill acquisition in the context of the *roda*—the setting where capoeira training takes place, with its concrete patterns of play, music, and bodily movements (Downey, 2005, 2012). Rather than viewing balance as innate, research of Downey showed how training, context, and interpretation shaped whether capoeira practitioners could overcome initial neural reactions such as the righting reflex while maintaining balance. In this case, understanding the specific outcome—balance—depended on a series of considerations that would not be available in current neuroimaging research but were highly relevant to how capoeira worked as a dynamic human skill.

Even these localized approaches to culture generally orient themselves within three broad approaches: symbolic and interpretive approaches, political economic approaches, and ecological and environmental approaches. Research can use more of an interpretive approach to culture, analyzing symbols, rituals, language, and meanings, as well as associated practices (Geertz, 1973; Tedlock and Mannheim, 1995; Holland et al., 2001). Or, one might look at how political economic structure shapes identity, sense of self, gender, class, consumption, and even disease (Mintz, 1986; Di Leonardo, 1991; Farmer, 2004). These social structures shape who we are, how we think and feel, our daily experiences, and the material resources we have to solve tasks and grow and develop. One could also take an ecological/environmental approach, with its emphasis on how ecology shapes ways of living, and thus ways of relating to nature and to others, as well as how one feels and thinks about the local environment (Biersack, 1999; Stepp et al., 2003; Zarger, 2010). Table 2 provides a way to help figure out which of these three major approaches to culture—interpretive, political economic, or ecological—will be the most useful for a research project that aims to assess the specific effects culture has within these domains.

**Table 2:** Cultural Approaches.

**Cultural Approaches**	**Potential Features**
1. Symbolic & interpretive	Symbols, language, ideology, religion, emotion and self
2. Political & economic	Inequality, political institutions, capitalist or egalitarian ideologies
3. Ecology & environment	Local ecologies, forms of food production, animal-human interactions, learning about nature

*What kind of cultural approach is useful for your research? Option 1: Does your research connect to symbols, language, ideology, religion, or something similar? Option 2: Does your research connect to economy, inequality, political institutions, capitalist or egalitarian ideologies, or something similar? Option 3: Does your research connect to the local environment or ecology, learning about nature, interacting with animals, or something similar? Option 1 is Symbolic and Interpretive, option 2 is Political Economy, and option 3 is Ecology and Environment (see Table 1). If you answered no to all three of these, consider a following option: Globalization or Material Culture. However, globalization and material culture approaches generally draw on one of the three main orientations—symbols, political economy, or ecology—for help in understanding specific aspects of how a changing world marked by migration, money, and media (globalization) or how the physical and interactive aspects of built environments, from clothing to smart phones to architecture to trees to reliquaries (material culture), relate to how people live, experience, and understand. Examples of Symbolic and Interpretive Research: Shweder's (1991) text is foundational to this line, looking at emotion, self, and morality across culture and centered on the person in relation to culture as a key analytic unit (see also Markus and Kitayama, 1991; Cole, 1998; Nisbett et al., 2001). Continued work in cross-cultural psychology (e.g., Bender and Beller, 2016; Sternberg, 2017) links cognitive phenomenon such as perception to culture, while a great deal of psychological anthropology continues examining culture in relation to Shweder's classic concerns with emotion, self and person-centered analysis (Quinn, 2018; Bock and Leavitt, 2019). Examples of Political Economic Research include: The psychology of neoliberalism (Adams et al., 2019), Decolonizing community psychology (McNamara and Naepi, 2018), Sense of closeness and helping behavior (Hackman et al., 2017), Nationalism (Sapolsky, 2019), Distributed cognition and economic reform (Lieber, 2015), and Folk-economic beliefs (Boyer and Petersen, 2018). Examples of Ecological and Environmental Research: Cultural neuroscience and psychology have looked at how patterns of food production, whether more collective (rice farming) or individualist (individual farmsteads), have led to changes over time in psychological and neurological patterning (Chiao and Blizinsky, 2009; Talhelm et al., 2014). Here, patterns of ecological productivity create both selective effects, favoring certain gene types over others, and enculturation effects, working through value systems, patterns of social coordination, and the demands of making a living. But this broad level is not the only way to connect culture to mind and brain. For example, research on how children learn about local ecologies emphasizes language, institutions like schools, family networks, exposure and experience, and other more immediate factors that shape how children think about nature (Hermann et al., 2010; Zarger, 2010; Quinlan et al., 2013; Ojalehto and Medin, 2015). Globalization and Material Culture: Globalization offer another major way to analyze cultural change (Andepson-Fye, 2003; Kirmayer, 2006; Hong and Cheon, 2017). Similarly, materiality—or looking at how the material aspects of our lives are central to cognition and culture—represents an important recent area of recent research (Hutchins, 1995; Clark, 2006; Sutton, 2008)*.

### Considering Cultural Variation

Three common issues affect “sizing culture appropriately,” or taking into account considerations of similarity and difference in cross-cultural variation. First is the WEIRD problem (Henrich et al., 2010) that most psychological research has been done with university students. These samples have been both homogeneous and skewed; these students represent not just Western, educated, industrialized, rich, and democratic but also young, privileged, and often self-indulgent and myopic (Lende and Downey, 2020). WEIRD results do not generalize well, and the antidote is not simply better replication of existing theories based on WEIRD samples (Muthukrishna and Henrich, 2019). Rather, research needs to recognize how context-dependent samples affect both results and the interpretation of results; research that makes broad assertions about culture and human variation must be examined in a comparative approach that takes into account the extraordinary range of variation already known to anthropology.

The second issue is that culture is a shared problem rather than an individual variation problem (LeVine, 1984). Many approaches in neuroscience and psychology focus on an individual as the unit of analysis, and use statistical approaches that emphasize how individuals vary from each other. Cultural science must move beyond treating culture as just another individual variable to measure (which often reduces culture to quantitative demographics, whether that is gender, world region, race, age, or similar type of measurement). This approach to “sizing culture appropriately” gives culture the theoretical space to be an actual working entity in the lives of the people participating in any particular study.

In anthropology, culture is generally approached as a shared phenomenon, for example, the shared meanings that a group uses to understand its place in the world. Take language. Psychologists might focus on variation in linguistic skill (say, evaluating individual reading ability) while anthropologists might focus on how the acquisition of literacy by a group creates broad changes in how people communicate with one another. Put in statistical terms, rather than studying normal distributions, anthropologists study J distributions, where most people share in a common culture (LeVine, 1984). An important research consideration related to the shared aspects of culture is that smaller samples become viable (Guest et al., 2006), since commonalities will occur in most members of even small samples.

The final consideration is to recognize the shared production of difference within members of a particular group or culture. In other words, shared culture can, nonetheless, produce a difference in ways that are not reducible to individual differences. Gender, class, and age represent recurring features of social organization, and, in turn, these social features shape what one does and knows. While these variables are often reduced to individual-level features in WEIRD research, they actually represent patterns of shared experience, behavior, knowledge, interpretation, and/or language.

### Individuals and Interactions

Given how patterns of shared experience form part of human variation, neuroanthropology uses an approach that considers individuals and interactions in relation to culture. For example, addiction has cultural and social roots—drug-use experiences are intimately related to symbols and meanings, while addiction runs along the fault lines of society, both at the level of households and of socioeconomic organization. Nevertheless, addiction can also be potentiated by certain types of interactions that happen in specific times and places (for example, hanging out at a “hard drinking” bar—Alasuutari, 1992) as well as whether drug use has sensitized the dopamine system (Lende, 2005). Thus, understanding addiction requires putting together how individuals interact with drugs as well as with local patterns of inequality, people, and places that favor drug use, and cultural meanings surrounding excessive use (Lende, 2012).

Similar to “sizing culture appropriately,” the level of granularity can matter when considering an individual and interactions (Table 3). For the individual, one can go from a focus on biology to psychology and then self and social self. At the biological level, one might consider not just brain function (Roepstorff et al., 2010) but also physiological functions. For example, Worthman (2009) argues that “habits of the heart” (affective-cognitive processing) shape individual relations toward immediate sociocultural contexts, and thus what local niches individuals sought out and how they worked to exploit social and material resources.

**Table 3:** Analytic levels: individuals and interactions.

**Level**	**Individual**	**Interaction**
Body	Biology	Embodiment
Mind	Psychology	Practices
Micro	Self	Context
Macro	Social Self	Social Structures

*Alongside sizing culture, researchers should consider the levels of analysis that they take with regards to the individual and interactions with culture. Culture can shape biology, psychology, self, and social self, but at the same time, these levels can change the impact and function of culture, particularly at meso, micro, and mental levels (see Table 1 on Sizing Culture). At the same time, interactions mediate between individual and culture, and thus can be actively considered in neuroanthropology and other research on culture, mind, and brain. The interaction level focuses on how culture can specifically impact the individual, from shaping physical bodies and biological functions via culture (Gravlee, 2009; Redcay and Schilbach, 2019); how social practices shape what people learn, how their biology functions, and how they relate to others (Downey, 2010; Roepstorff et al., 2010; Hari et al., 2015), how specific contexts shape how the brain interprets local information as well as what sorts of practices a person might use and what sort of self and social self they might present (Moore, 2004; Quinn, 2006; Schilbach et al., 2013), and how larger social structures can have quite individual impacts (Lende, 2012) as well as shaping context, practices, and embodiment (Gravlee, 2009; Roepstorff et al., 2010)*.

For self, one can draw on the discussion of self of Quinn (2006) in anthropological light. She argues that “self” references neurological and psychological processes but should be understood as a larger unit, one that can also exist in relation to cultural symbols and have contextual features shaped by interactions with others. Self, in this case, might be understood as a sense of identity and other types of “coherence” (in the words of Quinn) that brings together disparate aspects of experience into the sense of who an individual is as a person (see also Galloti and Frith, 2013).

Finally, self can also exist socially, for how we play certain social roles and present ourselves to others. This social self represents two different things: (1) the overlapping aspects of who we are, and how race, gender, class, and sexuality, as forms of similarity and differentiation that intersect within a particular person, shape a person (Crenshaw, 1989; Brah and Phoenix, 2004; Veenstra, 2011; McCormick-Huhn et al., 2019), and (2) how that intersection is not simply imposed but also agentive and performed, enacting who we are through our behaviors in specific settings (Butler, 1993; Barad, 2003). This acting in the social world comes from “a regularized and constrained repetition of norms” (Butler, 1993, p. 95), producing a certain social self, such as cisgender man, and precluding others.

Interactions, for neuroanthropology, generally focus on granularity that holds an individual constant rather than reaching down into how environment-organism interactions might shape things like epigenetic regulation, gut microbiomes, and similar aspects of how context can drive biology. At the broadest level, interactions between individuals and their local environments are structured by local socioecologies, which include how political economic factors shape neighborhoods (Chen et al., 2015, 2019; Nadan et al., 2015), scaling effects that happen in cities (Bettencourt et al., 2010; Turchin et al., 2018) and how social structures, in turn, shape how people learn class and other relevant aspects of social position (Mintz, 1986; Bourdieu, 1987).

One way to understand these interactions is to use the social science approach to practices (Bourdieu, 1977; Ortner, 1984), among others. A study of Downey has adapted practice *via* the notion of skill. Enculturation can work through skill, not just abstract knowledge and language, indicating the need to look at “changes in physiology, perception, comportment, and behavior patterns” (Downey, 2010, p. S22), where learning by doing and processes of embodied feedback shapes the creation of shared skills. A similar approach is in the “patterned practices” of Roepstorff et al. (2010) where social interactions can correlate with neural and psychophysical patterns. Here, looking at specific patterns of practice, whether in the field or experimental settings, moves researchers beyond more abstract notions of culture.

### Interactive Elements

Ritual provides an example of how practices use interactive elements that bring neurobiology and culture together. At a broad level, cultures structure ritual (Boyer and Liénard, 2006; McGraw and Krátký, 2017; Hobson et al., 2018). Rituals work because human groups create both particular settings and practices that, in turn, can drive human biology *via* specific processes that interact with a person, from the social self to basic neurobiology. Indeed, cultures have figured out that aversive rituals can produce group cohesion through intense events, whereas routine rituals work through repetition (Whitehouse and Lanman, 2014).

Researchers such as Rappaport (1999) and Turner (2011) have highlighted how rituals are seen by individuals as compulsory yet work through the condensation of specific elements that can produce subjective and neurological changes in participants. Rituals work by combining sensory elements with associative elements (or ideological elements, in the terminology of Victor Turner), focused on specific material instruments and bodily practices within the ritual (Bull and Mitchell, 2015). These interactive elements produce specific types of neurological effects, for example, blurring self/other processing, extending the tendency of humans toward immediate social rewards to broader sociocultural elements, extending associative learning to capture aspects of meaning through the concentration of linked associations, and using intense experience to promote neural reuse *via* cross-talk (as psychedelics have long done; Xygalatas et al., 2013; Whitehouse and Lanman, 2014; Bull and Mitchell, 2015).

Interactive elements are not the same as affordances, the concept initially devised by Gibson (1986) to refer to aspects of the environment that can support and even help accomplish cognition. Recently, affordance approaches have been applied to culture (Ramstead et al., 2016; see also Lende and Downey, 2020, for a discussion). While interactive elements might be seen as comparable to affordances, these interactive elements are conceived first from the point of view of culture in relation to an individual, not from an internal position of mind or cognition.

The materiality and patterning of the interactive elements that operate between individuals and culture are central to how culture has figured out how to hack the brain. They are, in many ways, analogous to the material processes that mediate between DNA and cell function. These interactive elements are another set of mechanisms, just like epigenetics. They are interactive elements on top of our cognition, and thus not reducible to affordances that exist in relation to cognitive processing and biophysical constraints. Crucially for research, these interactive elements are there in what people say and do, not just in the biological and cognitive processes that comprise the lower levels of individual analysis.

## Triangulation

### Conceptual Triangulation

A central concern for interdisciplinary research on mind, brain, and culture is how to productively bring together different ideas and methods. While there are many ways to do this, we have found that triangulation proves useful. In its initial use, triangulation meant using multiple methods to produce a more complete picture of the phenomenon under study while also counteracting, *via* those same multiple methods, the biases that come with relying on any one data source (Patton, 1999; Maxwell, 2004; Denzin, 2010; Fusch et al., 2018). Today, triangulation also applies to combining different theoretical viewpoints (Pitre and Kushner, 2015; Fusch et al., 2018). In our work, two types of triangulation help to frame research: laboratory-clinic-field and psychology-neuroscience-anthropology.

**Figure 1:**
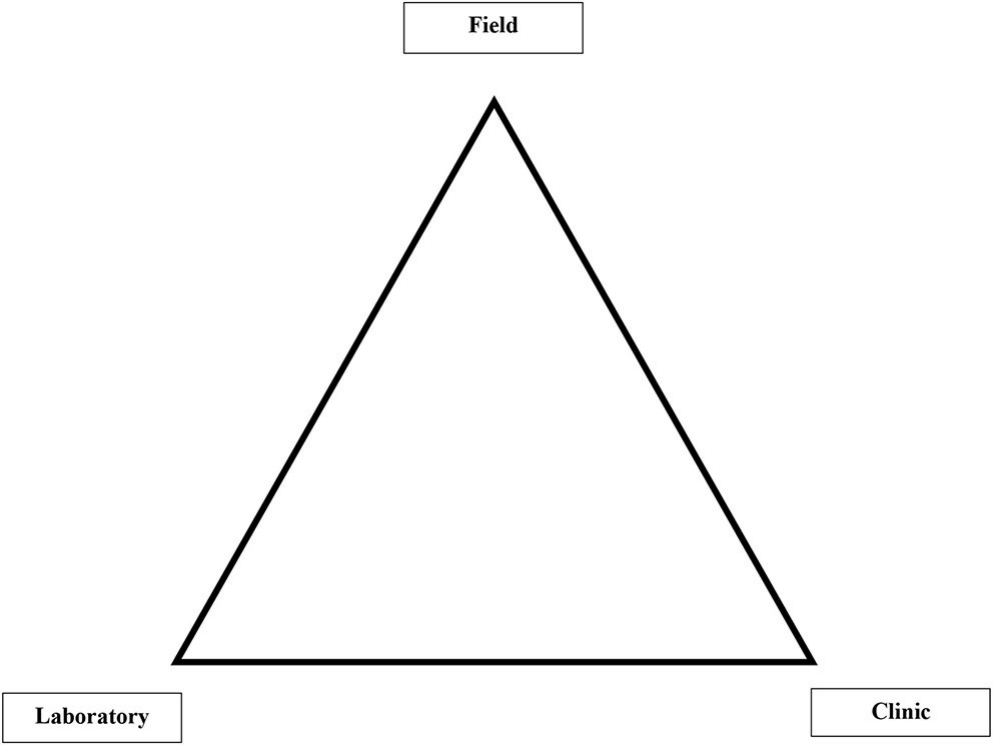
Conceptual triangulation between field-laboratory-clinic. Conceptual triangulation happens *via* asking questions and considering the data and viewpoints from different arenas of scholarship. For example, how can field research test ideas that come from clinical and laboratory based settings? What results and ideas do they offer that are relevant to what I am finding? What is lab-based research missing that is apparent in the field? How does the clinical problem present itself in everyday circumstances? What other factors shape this problem in the everyday that are apparent in the field but might not be in the clinic? The onus is on the field research to, first, understand research from laboratory and clinical settings, and second, to look at how one can provide data that can at least inform a scholarly debate. One might also consider what bridging ideas exist between field-clinic and field-laboratory. Where is the common ground? What specific approaches can mutually inform each other? What are specific methods, research foci, and theoretical issues that overlap between field-clinic and field-laboratory? For further ideas on integrating field and laboratory research, see Willis and Miller (2011), Teddlie and Tashakkori (2012), Hay (2016), Gurven (2018), Hruschka et al. (2018), and Matusz et al. (2019). For field and clinical research, see Weisner and Hay (2015), Kirmayer and Ryder (2016), Kaiser and Kohrt (2019), Lewis-Fernández and Kirmayer (2019), and Watson et al. (2019).

Laboratory-clinic-field references how laboratory, clinical, and field research can all add to understanding a problem (Figure 1). Neuroanthropology, with its field-based approaches, can provide a crucial empirical check on theories and results that come out of laboratory and clinical settings. This research is often presented as helping to understand how a specific problem plays out in the “real world” but generally does not take the additional step of testing its ideas in that real world. At the same time, field-based research should aim to develop data that can address relevant issues and ideas that come from the clinic and the laboratory. Certainly, researchers using neuroanthropology can address theoretical and practical problems that emerge primarily from field research. But these data do not have to exist on their own; developing conceptual triangulation increases the relevance of field-based data to researchers and practitioners in the lab and clinic settings; Figure 1 provides further suggestions.

The second type of conceptual triangulation is psychology-neuroscience-anthropology. Neuroanthropology started with an emphasis on the intellectual yields that come from integrating neuroscience and anthropology. Cultural neuroscience performs a similar intellectual move, focusing on psychology and neuroscience (Kim and Sasaki, 2014; Chiao et al., 2016). Substantive integration of all three disciplines is the logical next step, although often difficult in practice. As a convenient shorthand, this type of triangulation can start from a simpler premise: research should address mind-biology-culture.

Mind is not uniquely the domain of psychology, as there are many disciplines that consider thought, affect, subjective experience, and more. Similarly, biology is not unique to neuroscience; biological anthropology, evolutionary biology, comparative biology, and physiology all explicitly consider biology, whereas social science and humanistic inquiry increasingly address notions of the body, embodiment, corporality, and materiality. Finally, culture long ago escaped anthropology, and is now considered in many disciplines, including in evolutionary biology. Thus, mind-biology-culture represents a pragmatic approach, ensuring that each domain is explicitly considered during the development of research.

### Critical Complementarity

Simply stating that triangulation is useful is not sufficient to indicate how to actually triangulate approaches that can come from disparate fields with differing types of data, theoretical assumptions, and scholarly emphases. Triangulation is inevitably strengthened the more it can be tied into research that comes directly from psychology, neuroscience, and anthropology, and even better, placed within the inevitable disciplinary debates within that field, rather than assuming that the insights from another discipline are somehow less laden by constraints of methods, relevant data, disciplinary debates, and intellectual history.

Here we advocate using “critical complementarity” as a mechanism to figure out what is useful for robust research on the interaction of culture and people versus what might remain discipline- or site specific. Critical complementarity uses critical analysis first to understand background assumptions and methodological biases, and then stresses how different ideas can complement each other, providing their initial limitations are taken into account. Critical complementarity brings together the strengths of critical approaches with the interdisciplinary emphasis on finding which ideas and data can best illuminate a particular problem.

Here, it is easiest to discuss the research of Lende (2005) on incentive salience and addiction. The incentive salience approach to dopamine function represented a cutting-edge theory about what drives addiction (in a nutshell, incentive salience mediates “wanting” *via* dopamine signaling, and drugs drive excessive salience signaling, thus heavy users want too much and use to great excess). However, this research had two critical limitations: (1) it was laboratory-based research, using animal models, and thus not tested with humans in either clinical or field settings at the time, and (2) it advocated strongly for its particular view of dopamine function against other interested actors in neuroscience and thus did not fully consider how incentive salience might usefully combine with other types of dopamine-related research.

At the same time, there was complementarity between what Robinson and Berridge (1993) proposed as the subjective experience of incentive salience—wanting—and views of Colombians of addiction as a problem of “wanting more and more” (quite different from the emphasis in the United States on pleasure). Yet some anthropologists would take this cultural view of “wanting” and assert that dopamine function is completely constructed *via* culture; a critical view from biocultural anthropology argued against reducing the subjective experience of desire to either culture or biology (Lende, 2005). Finally, the approach of both Robinson and Berridge and cultural anthropology to “wanting” left it rather undefined as a psychological process. Rather than drawing on a specific psychological theory, this research drew on experience-near and person-centered approaches in anthropology (Wikan, 1991; Levy and Hollan, 1998). Intensive questions helped informants to describe both specific experiences and how they felt and acted in specific contexts; this qualitative understanding of wanting then informed the development of a scale to test whether incentive salience was actually reported by people who had greater problems with drug use and abuse.

For guidance on the neuroscience side for how to do critical complementarity, one can combine critical neuroscience (Choudhury and Slaby, 2016) with cultural neuroscience (Chiao and Ambady, 2007; Kim and Sasaki, 2014; Kitayama et al., 2019). Developing critical complementarity also means drawing on social science research, for example, critical analyses done by anthropologists and others on neuroscience (Martin, 2010; Pitts-Taylor, 2010; Rees, 2016) as well as biocultural and integrative approaches (Hruschka et al., 2005; Seligman and Brown, 2010; Fitzgerald and Callard, 2015). The point is not to rely excessively on a particular perspective but to use critical complementarity to build bridges among different areas of research.

### Methodological Triangulation

By using a combination of methods, researchers can address differing aspects of culture, examine how individuals interact with culture, and capture aspects of say-do-process as relevant to the research problem. Given the emphasis that neuroanthropology places on the field, ethnographic methods are often important. Ethnographic methods attend to the perspective of the participants in the field, capture context effectively (particularly through participation as well as methods that let informants show what contexts are like, such as Photovoice), and buttress the ability for researchers to understand how people engage in symbolic interpretation. Similarly, qualitative methods can permit rapid assessment of results from laboratory, clinical, and epidemiological studies—claims that results reflect such and such among a study population can be assessed, using rapid ethnography, focus groups, and expert interviews. Within mixed methods approaches, triangulation increasingly means addressing the applied and societal implications of research to increase its impact and relevance (Fusch et al., 2018).

At the processual level, different methods are useful. For example, between psychology and anthropology—or what people say—methods that rely on the language are preferred, from studies of metaphor and embodiment on the anthropology side to how language can work between sensory perception and sensory discrimination, for example, with olfaction. On the anthropology and neuroscience side—or what people do—mobile methods can be particularly effective, whether these are participant observations by the researcher (such as focal follows), intensive interviews that elicit what people do during the course of a particular day or event, and technological assessments that permit the capture of biological and psychological data as people move about and interact in and across settings.

Finally, on the neuroscience and psychology side—or what people process—one can use a variety of approaches. First, mobile psychophysiology increasingly permits real-time assessment of ongoing bodily reactions, potentially reflecting underlying processes. Second, biomarkers—for example, blood spots that can assess a range of biomarkers or saliva that can assess stress reactions—permit an assessment of accumulated processes, and thus insight into biological processes that might not be available using other field-based approaches. Other approaches in this vein include elicitation techniques and quasi-experimental techniques that can combine aspects of controls and comparative research with specific results.

But these types of experimental and quantitative methods are not the only ones useful to get at process. Qualitative methods serve equally as well, provided they take into account considerations already raised in this paper. For example, intensive interviews can address process—for example, incentive salience and how it relates to drug use—provided that the interviewer focus on specific processes and moves beyond just getting informants to describe what people say. In other words, people often give pat answers rather than getting into the nitty-gritty of how something actually plays out for them.

Participant observation methods can also address what actually happens at the processual level. Here, the researcher can take informants at face value (yes, capoeira actually changed how my balance worked, inside and outside the roda—it is not simply “culture” saying that) as well as using neuroscience to attend to changes that relate to underlying processes, for example, how the righting reflex when one falls backward gets relaxed in capoeira. In this case, methodological triangulation involves bringing together neuroscience of process, descriptions of change related to practice and context of the informants, and the match of the experience of the researcher to both the neuroscience and the explanation of the informants from their own points of view.

This long-term inductive approach centers the validity of the data in real-life settings, in watching and talking about how things play out in local lives, and, from there, creating a dialog with theoretical approaches. Rather than using the lab or clinic to fix a theoretical perspective, ethnographic work aims to discover patterns in the data and then find corresponding ideas for deepening analysis.

## Approach to Case Studies

Together, the nine elements provide a holistic approach that sizes culture appropriately and uses effective triangulation of theories and methods. The case studies show how these elements come together in specific projects and illustrate three important takeaways for this type of research: (1) do field research because that matters for understanding specific problems, (2) do not rely on just anthropological or neurobiological approaches to get at what happens—complex problems require critical complementarity, and (3) get types of data that help assess which combination of theories makes sense of any particular problem.

## Example #1: Brazilian Jiujitsu and Veteran Reassimilation

In research between 2015-2018, co-author Collura worked with a group of 20 U.S. military combat veterans to understand their assimilation back to civilian life. In particular, he examined how their participation in Brazilian jiujitsu (BJJ) assisted reassimilation. This research recognized that veterans had to negotiate dual demands: (a) entering the armed forces and going to war were processes of enculturation with their own social structure and reward system that shaped desired outcomes and interactions within combative environments, and (b) returning to civilian life brought its own challenges, from navigating a cultural environment structured differently than the military and the loss of the rewards and interactions from the military. For veterans, transitioning to civilian life post-military service also did not have the same institutional support—whether military or civilian—to facilitate the enculturation that they encountered when they enlisted (Finley, 2011; Collura and Lende, 2012). On both sides, the US cultural assumption is that, as individual adults, they had to handle this process of change on their own. In contrast, societies elsewhere often provide specific institutions and cultural ideas that support adult men as they transition to new roles in their lives (Evans-Pritchard, 1937; Wood, 1999; Worthman, 2010). This research looked at what particular individual and institutional elements of BJJ made the difference in the reassimilation of veterans.

Research included participant observation doing BJJ with veterans and interviews on military life, transition to civilian life, and BJJ. Methodologically, this research took a close-in view of culture, sized at the level of the training academy, the mat where participants grappled, and the social interactions between the men. The research also emphasized an experience-near understanding of psychology rather than utilizing measures to assess psychological states such as trauma. The combat veterans expressed considerable hesitancy and even distrust about these assessments; thus, the scales initially planned were left to one side as a way to heighten validity through increased ability to interact freely with the veterans. Finally, the research drew on neuroscience to go beyond “ethnography as usual” by also paying attention to task demands, stress, neuroplasticity, and learning.

The research revealed an ethnographic model around the engagement of veterans in the military, their transition to being veterans, and why BJJ helped with that transition. This model highlighted the importance of physical + mental transformations to enculturation, often grounded in how basic training had impacted their lives and, from there, recognized how much the social side of military life had shaped how they became accepted and valued members of a group. Thus, the veterans used a mind/body model located within an individual (D'Andrade, 1987) but also stressed how the social mattered in negotiating challenges (Quinn, 2006).

By shaping how individuals interpret what happens to them, such cultural models can impact how the function of their nervous system (Quinn, 2006; Lende and Downey, 2012a). For combat veterans, “physical + mental and social” referenced combining the mental side of a task with the physical side in order to achieve a specific outcome in defined contexts. This model was drilled into them time and again; the combat veterans saw the power in their ability to deal with the real-time ebb and flow of powerful sensations while having to cognitively filter what needed to be done at any given time.

**Figure 2:**
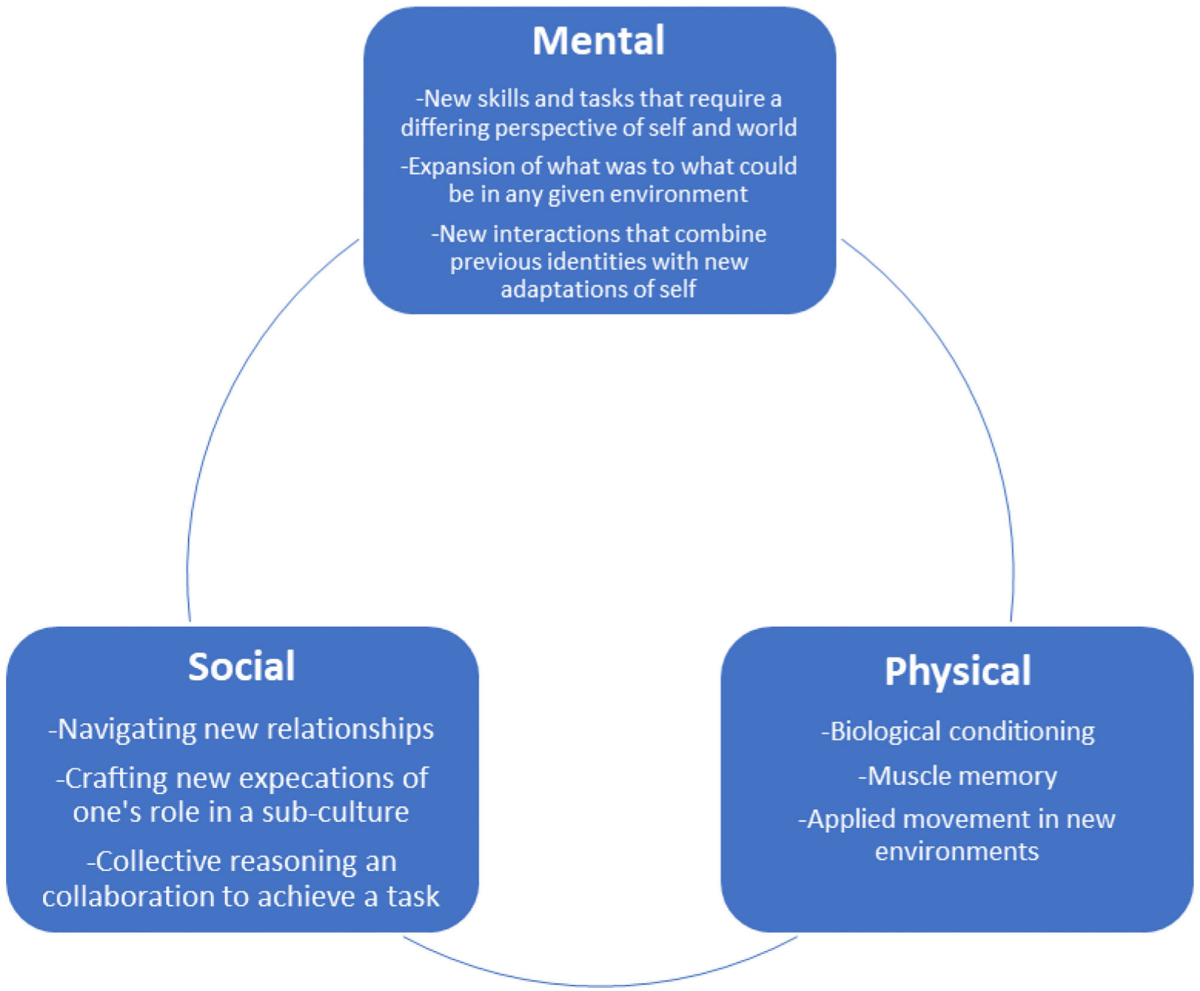
The physical-mental-social model as it played out in Brazilian Jiu Jitsu (BJJ) with military combat veterans. BJJ helped with the transition to civilian life because it engaged all three elements, and relied on how the men talked, practiced, and experienced BJJ. It is important to note that any activity during training could start as social, physical, or mental and mesh into the other elements. For example, the new social circles of the academy can also bring mental models that require physical interaction; at the same time, new physical activity challenges previous mental models and push individuals to new social interactions.

In subsequent analyses, we adapted this ethnographic model to understanding why BJJ proved effective. This approach—working with an informant model to inform subsequent theory development—was inspired by Downey (2005, 2012), where a “native model” of how balance changed while becoming a capoeira practitioner integrated physiological changes with how specific training practices mattered. For veterans, they found that BJJ training was very similar to the “physical + mental and social” model that they learned while going through boot camp, specialty schools, and combat deployment. Using neuroanthropology, we modified their ethnographic model of “physical-mental and social” to a physical-mental-social model (see Figure 2), which recognized how each element can flow into the next.

These different elements combined to provide an effective task structure for combat veterans. Overall, BJJ required veterans to negotiate intense conditioning that had specific goals. This type of practice worked best when the cognitive side of participation was in sync with the physical side and *vice versa*. For example, not hurting your partner while engaging in combative play was central to BJJ practice, demanding that embodied practices work in line with specific rules and objectives. As veterans built aptitude in a specific BJJ task, research showed simultaneous growth between mental ability, physical prowess, and social relationships.

Overall, BJJ combined different types of task structures into one encounter. First, BJJ requires the synchronization of bodies and thus parallels how coordinated behavior in laboratory settings involves inter-brain synchronization handled by fast, nonmentalizing neurological systems (Dumas et al., 2010). Second, BJJ involves cooperation, where opponents play within a set of rules and have to modify ongoing actions and responses. At the same time, BJJ is a competitive social encounter, looking to make an opponent submit. In task-based studies, social coordination and competition involve different neural systems (Decety et al., 2004; Nummenmaa et al., 2018) to negotiate these tasks. In summary, BJJ provides a complex task structure that brings together synchronization, cooperation, and competition in ways that require the activation and coordination of brain systems in real time.

Furthermore, it was clear during the research that BJJ also got “under the skin.” During training, physical exhaustion was obvious, given the sweat dripping off their bodies. Given that BJJ was similar to physical training in the armed forces, it provided an acceptable way to engage in strenuous activity that helped reduce stress and blunt the reactivity they often developed while deployed. This type of intense training has been linked to the promotion of neuroplasticity and thus likely has carryover effects on helping these veterans reassimilate versus those who do not exercise (El-Sayes et al., 2019). Moreover, almost all participants expressed a need for something to “cut the edge” from everyday civilian life. For some participants, the comfort and solace found in their BJJ training also provided a way to actively avoid illegal or prescription substances because they knew they had training the next day or because their need to get into better physical condition superseded their longing to use.

When navigating daily stressors and adversity, training in BJJ helped the military veterans frame assimilation stressors in a more productive context. In the BJJ academy, the hierarchy, the physical sacrifice, and the demanding nature of the training took the participants back to a familiar environment that helped them express and change their understandings of violence and trauma. For example, BJJ required intimate physical contact while requiring them to be in control, thus helping to rework their understanding of what combat and restraint meant. Moreover, the physicality of the BJJ experience and the acquisition of new skills often brought a new perspective on one's self, but that happened *via* negotiating relationships on the mat in ways that were both experienced and interpreted in ways quite different from what happened in the military settings. Returning to Wilson and Golonka (2013), BJJ provided an extended task structure that involved their bodies, partners, local meanings, and social structures, thus directly linking an internal view of the tasks the brain had to solve with the agentive and cultural ways that these veterans engaged with BJJ.

Overall, this research revealed that successful assimilation is helped by drawing on current and prior experiences to reframe how veterans can and should act. These are conceptualized as cultural bridges, where similarities in cultural practices and meanings can help individuals adapt. In BJJ, familiar notions such as a uniform, structured goals, and a sense of collectivity and belonging signified by material culture proved useful to participants. BJJ used similar components of self, social self, and contextual interactions but within an environment that was both new and welcoming to the men and their families and friends. This applied impact resulted from bringing together neurological function, learning, and plasticity with a task structure in a dynamic and locally constituted context.

Veterans then were able to link these changes with successful management of other areas of their life. Cultural bridges—rather than individuals finding their own way—can help people take intense enculturation experiences and reapply them to areas that are not directly part of the initial enculturation. For the veterans, BJJ served as a bridge activity from military to civilian life, helping them to explore differing mentalities and identities while engaged in both physical and social interactions. The effectiveness of cultural bridges from one domain to another likely plays a role in similar intense activities, such as dance and its effect on diminishing depression (Pylvänäinen and Lappalainen, 2018), how playing an instrument can decrease autism severity (Broder-Fingert et al., 2017), and how being balanced helped capoeira practitioners in other domains of their life (Downey, 2005).

For future research, it would be useful to incorporate biomarkers into the research (Worthman and Costello, 2009). In particular, cortisol measures might show differences in diurnal patterns of activation on BJJ practice days versus non-practice days, providing a measure of biological stress processing. Building on work on storytelling and brain synchronization (Hasson et al., 2012), BJJ practitioners could jointly recount a story about a recent bout while being scanned, thus providing insight into the neural dynamics of BJJ as a coordinated activity.

## Example #2: Raptors, Interactions, and Culture

Since 2016, co-author Hoyt has investigated human-raptor relationships, looking at how these relationships are formed and examining what processes make raptors such good candidates for use in animal-assisted therapies. In the past few decades, animal-assisted interventions (AAI) have developed alternative forms of therapy that use animals as “emotional mediators” and “catalysts” for facilitating improvements in health and well-being (Kulick, 2017). While dogs and horses constitute the majority of AAI treatments, employment of other species, such as dolphins, cats, and smaller farm animals, have become popular additions. Conversely, therapies involving raptors are far from the mainstream. Raptors are the antithesis of “cute” and “cuddly” pets that comprise the majority of human-animal bond research. Working with raptors requires considerable attention and patience, as one misstep could mean the difference between a successful training session and a spontaneous trip to the ER. Yet volunteers who work with raptors claim to experience similar therapeutic outcomes to those described in other animal-assisted approaches.

Our research looked specifically at a program in the southeastern United States that facilitated interactions between birds of prey who were not able to be released back to the wild and local volunteers. The research used a multispecies approach situated within ecological anthropology, recognizing that animals, plants, and the natural environment participate in the formation of culture (Haraway, 2003; Kohn, 2013; Rees et al., 2018). This multispecies approach combined with neuroanthropology to provide a framework to examine the assemblage of social, environmental, and neuroanatomical pathways that connect us with other species. Specific methods used in the research included focus group discussions, semi-structured interviews, and over 150 h of participant observation. Alongside assessing the program as a whole, research specifically engaged the participants who self-identified as suffering from trauma and stress. These participants indicated that working with raptors improved their ability to manage their daily lives. Field-based research of Hoyt focused on examining how and why raptor therapy made a difference.

Research on raptor cognition has long had to deal with culturally embedded ideas about animals, particularly higher versus lower animals (e.g., “bird brains”; Shimizu, 2009). For example, the presence of a six-layered neocortex in mammalian brains not seen in birds was long thought to afford the ability to perform complex computations and behaviors only in mammals. In fact, avian brains are capable of performing highly complex tasks, including remembering the past, reasoning about how to manipulate objects and thinking about perspectives of others (Clayton and Emery, 2015). Yet birds use a different neural architecture that relies on an alternatively designed nuclear pallium and an enlarged optic tectum (Shimizu, 2009). Avian brain studies have also helped to debunk myths like “bigger is better.” Brain-body scaling techniques focused on neuronal densities, rather than relative brain size, have revealed how ravens possess 1.2 billion more pallial neurons than capuchin monkeys (Olkowicz et al., 2016). Based on this avian research, we approached raptors as capable of complex cognition that is both responsive and situationally dependent. Drawing on the systemic approach of Hutchins (1995) to cognitive tasks like flight and navigation, we viewed birds as shaping both the larger systemic context and the specifics of interactions between handlers and raptors.

Our research first explored the infrastructure of the nonprofit raptor program to better grasp the larger elements involved in bringing people and raptors closer together; these elements formed the dynamic backdrop through which raptor-human relationships developed. Data indicated the importance of “charismatic leadership” and “sense of community among volunteers” for the program. The director provided guidance, wisdom, and motivation for volunteers through continual program expansion, generation of new ideas, passion for birds, and willingness to offer advice whenever necessary. A strong sense of “volunteer community” played a significant role in retaining long-term volunteer handlers through the formation of friendships and shared interests in raptors that did not exist outside of the park. Thus, the reasons for volunteering with the raptor program ran deeper than simply interacting with the birds; the experience provided a chance to spend quality time in nature with other “bird nerds.”

Humans have cultivated relationships with raptors for at least 2,500 years (Epstein, 1943; Oggins, 2004; Soma, 2012), making these relations part of the novel multispecies cultures that emerged throughout the Anthropocene (Prummel, 1997; Ikram et al., 2015). However, relations with raptors are often different from those with mammals. As apex predators, raptors possess a number of adaptations uniquely suited for spotting and killing an unsuspecting quarry. When dealing with humans, raptors can be dangerous, often temperamental creatures who can evidence significant disinterest and even distrust toward humans. Thus, it was not intuitively obvious why interacting with raptors helped the participants.

Over the course of the research, participant observation helped guide the development of an approach for understanding how multispecies relationships facilitate novel forms of regulatory processing. In 1992, Bagozzi called for an approach to self-regulation that addressed the “processes that occur between intentions and goal-directed behaviors” (Bagozzi, 1992, p. 200). Argument of Bagozzi against reductive terms like “attitude” resonated with holism of anthropology, and his proposal that both intentions and behaviors matter for self-regulation provided a way to understand that raptors and people were actors within a regulatory network of any person.

Building from critical complementarity to field research, our research focused on identifying the interactive mechanisms that happen in small-scale contexts between individual raptors and humans. Self-regulation, as a generic concept, discounts aspects of context and individual experience that proved important for human and non-human interactions observed during fieldwork; in turn, these interactions shaped whether human participants had positive or negative outcomes. These interactions are akin to the “hidden regulators” that Hofer (1994) described. Hofer viewed “attachment” as an overarching concept to understand specific physiological processes that played out between rat pups and mothers. These hidden regulators are identified through breaking attachment into smaller pieces—specific interactions and physiological processes then constitute the differential effects the mother can have on the rat pup.

This project recognized the need to capture data that were relevant to psychological and AAI approaches while engaging in participant observation. By moving between ecological validity (data to relate back to laboratory and clinical approaches) and ethnographic validity (data that capture how they interact in specific contexts, and examining that interaction from the point of view of both the bird and the handler), the research aimed to build a better model of what happens between birds and handlers.

By using neurobiology and psychology to help interpret the data, we identified six processes that existed in raptor-human interactions that could be linked to regulation. These were selective attention, modified response, physiological feedback, reward, novelty/threat, and resiliency. For example, “modified response” referred to how a bird of prey trainer could intentionally change his or her approach to accommodate the needs of the raptor he or she is holding; consequently, this behavioral shift afforded raptors the opportunity to return “physiological feedback” to their handler as the raptor equivalent to “thumbs up” or “thumbs down.”

However, bating behavior—when a raptor jumps off the glove—was often interpreted as a direct result of things, such as particular personality traits that make some birds less amenable to training, a lack of proficiency on the part of the handler, or an outright form of protest against living in captivity. The anthropomorphic associations at times limited multispecies interactions because nonhuman behaviors were viewed as either static and unchanging or responding only to human activity.

For example, “selective attention” mattered in the context of bird handling. If the person was training, he or she should be looking at the bird. This might seem obvious; however, raptors can sit still for long times, and some handlers interpreted the bird as calm and then started to look at other things in their local environment. By not paying attention, handlers might not notice that the bird could become fidgety. In such a case, “selective attention” could create waves that the handlers had to correct. In the context of a specific training bout, the bird jumping off the glove was not good. However, these unexpected reactions were generally good in the long term, because it added to the novelty of continuing to work together and finding a balance between the bird and the handler.

Overall, this project demonstrated the need for a two-step approach to understanding how interactions can impact regulation and therapeutic outcomes between birds and handlers. First, having an on-the-ground understanding of the institutions and specific contexts proved important. Second, getting at the processes that constitute interactions between raptors and handlers is crucial. **Figure 4** illustrates how a general model of raptor-human interaction then takes life with the specifics of ethnographic research and the neuroanthropology of interaction and regulation. Future research could bring in portable electrophysiology to measure arousal and engagement while participants interact with raptors.

## Example #3: From Cue Reactivity to the Neuroanthropology of Cue-Driven Drug Use

The final example covers research on cues and substance use currently in development. Cues have long been used as a way to investigate how individuals learn, from the dogs of Pavlov salivating at the sound of the dinner bell to rats pushing a lever to access a drug reward (Yokel and Wise, 1975). With addiction research, cue reactivity has formed a theory of relapse, which proposes that people can be “particularly vulnerable to drug use when in the presence of stimuli related to previous episodes of use” (Carter and Tiffany, 1999, p. 327). Over time, models for cue reactivity have moved beyond the stimulus-response paradigm, in which addicts are unilaterally drawn toward use by cues, because of changes in understanding how complex addiction and recovery are. But research on cue reactivity has also shown several limitations. First, research relating to cue reactivity and rates of relapse has produced mixed results; for example, physiological and subjective reactions to cues are not directly linked, and only physiological cues have been shown to be correlated with future rates of relapse (Witteman et al., 2015).

Another limitation is that cue reactivity has yet to yield significant clinical applications (Childress et al., 1993; Conklin and Tiffany, 2002; Mellentin et al., 2017). While there is some recent evidence of cue-based approaches having value-added clinical impact (Wiers et al., 2014, 2015; Kober et al., 2017), the most clinical practice still focuses on established therapies and on dealing with the consequences and family impacts that come with addiction. The lack of a match between lab results and clinical outcomes demonstrates the complex nature of the cue reactivity phenomenon.

Here, we draw on the integration of clinical and laboratory research of Drummond, which highlights three domains of reactivity: symbolic-expressive, physiological, and behavioral (Drummond et al., 1995; Drummond, 2000). The first domain is craving, or the reactive surge in wanting a cue such as seeing someone use. Symbolic-expressive cue reactivity represents the interpreted urge people feel when presented with an internal (stress, anxiety, etc.) or external (paraphernalia, advertisements, etc.) cue. The second domain is physiological cue reactivity, the bodily effects (increased heart rate, skin conductance, changes in temperature, etc.) that come from tolerance, opponent process reactions, and other physiological reactions that come with interaction with cues (Smith, 1990; Robinson and Berridge, 1993, 2008); physiological reactivity can happen both in anticipation of use and withdrawal.

Finally, behavioral cue reactivity comes *via* obtaining and using drugs; these actions in themselves can be cues that prompt relapse and augment use. On the seeking side of behavioral cue reactivity, the cue of the lever can drive lever pressing in laboratory animals; for humans, seeing alcohol might lead to an impulsive purchase and then relapse. On the consumption side, behavioral cue reactivity lends itself to excess, for example, the speed or overall amount of drinking. Pregaming on college campuses, where students get together to drink large amounts before going to a sporting event, is a social situation that can drive behavioral cue reactivity leading to extreme intoxication.

Overall, most of the research has looked at symbolic-expressive and physiological drug craving in a laboratory or otherwise controlled settings (Rohsenow et al., 1994; Carter and Tiffany, 1999; Conklin and Tiffany, 2002; Sinha, 2007; Carpenter et al., 2009; Verdejo-Garcia et al., 2012; Witteman et al., 2015). Recently, some behavioral research has aimed to bridge the gap between the lab and the field. For example, Witteman et al. (2015) utilized long-term journaling as a method of data collection, and Shiffman et al. (2008) used ecological momentary assessment (EMA) as a way to study cue reactivity in context (Shiffman et al., 2008). In these studies, participants employ mobile technology to do stimulus-response activities at intervals throughout a day (Warthen and Tiffany, 2009; Wray et al., 2015). However, these studies present cues specifically selected by investigators rather than naturally occurring environmental cues and generally assess the relationship between cues and seeking after the fact.

Despite these developments, cues remain undertheorized in terms of how they form part of the interactions between individuals and their local environments. Here, we view cues as forming part of the everyday, even mundane aspects of using and recovery. Cues can signal opportunities to use, to forget, and to do something different; as such, cues provide an adaptable framework to consider many different factors that can be important to facilitating and sustaining recovery. In this way, cues are amenable to ethnographic work, using a neuroanthropological lens. From the point of view of neuroanthropology, cue reactivity connects the brain, learning, and the environment.

Conceptual triangulation can first focus on the field-clinic-lab triangle. Given the experimental approach, cue-reactivity research, in general, cannot focus on actual moments of drug seeking and relapse; rather, cue reactivity has examined often artificial aspects of context (e.g., generic pictures of someone drinking or of liquor bottles). Thus, this research could benefit from triangulation that can provide field-based data on how cue reactivity might work in naturalistic settings. Conceptual triangulation can also pull together insights from anthropology, psychology, and neuroscience to drive forward a greater understanding of context-driven cue reactivity (illustrated in **Figure 4**). Neuroscience and psychology together help make clearer how people engage with their environment, how they attend to meaningful cues and the physiological components and outcomes of deep-rooted attentional bias.

Cue reactivity research sets the expectation that meaningful cues lead to craving, drug seeking, and, potentially, relapse. A neuroanthropological approach can consider biological and ethnographic variables and assess the relationship between physiological, interpretive, and behavioral craving and encounters with stimuli. In particular, an ethnographic approach can better capture the moments participants actually encounter cues and how they react in their everyday lives. For example, the incentive salience approach to associative learning and cues can also provide insights *via* neuroscience. According to this theory, cues—to be compelling—have to be imbued with incentive salience, or something that causes people to pay attention, move toward, and desire what is linked to that cue (Robinson and Berridge, 1993, 2008). However, what cues work better in real-world settings to capture the attention of people and to be the targets for greater attribution of incentive salience are questions that are hard to address, particularly, initially, in the lab.

Thus, anthropology, through its understanding of how contexts can funnel drug use and other activities like gambling (Schüll, 2012), provides a way to understand cues from the perspective of the context. This field-based work can also increase validity and potentially provide both theoretical understanding of how cues work in contexts as well as novel stimuli that can be more contextually and individually relevant. By understanding field-based dynamics better, we might be able to reinvigorate ideas and implementations of this research in clinical settings.

To make these insights come together will require methodological triangulation. First, by spending time in the local environments, ethnographers can capture instances of use and craving, when they occur, and the surrounding events (internal and external cues). From there, a combination of EMA research (real-time assessment of cues and craving), assessment of physiological reactivity (heart rate and electrodermal activity), and prolonged participant observation and/or interviews could look specifically at how to relate contexts and experiences to the types of considerations of cues and cue reactivity outlined above. Finally, participants could use photovoice (photos taken by informants on a specific topic, for example, what triggers a craving) to capture what cues look like for them in real-life settings, providing both prompts for subsequent interviews as well as personalized cues that could be utilized in a laboratory setting. Overall, a neuroanthropological approach to cue reactivity employs interdisciplinary theoretical and methodological innovations to better approach how cue reactivity can be applied in clinical settings.

## Conclusion

Ethnographic research in field settings often reveals gaps between laboratory research and the specifics of a particular problem in a naturalistic setting. The three examples above all used qualitative approaches to better bridge between how problems manifest and what is known from experimental research. This interdisciplinary negotiation between anthropological approaches to human variation and specific studies that tease apart the variables and processes that shape that variation is central to how neuroanthropology contributes to cultural science.

Each case study can also illustrate specific elements of the overall framework shown in Table 4 for how to engage in neuroanthropological research. For example, the research by Collura with veterans located the problem of the veterans at the intersection of biology and culture. What the veterans faced was not simply an individual problem, for example, PTSD or a lack of coping. As combat soldiers, they had been encultured into the military; becoming veterans was also an encultured transition.

**Table 4:** The elements of neuroanthropology.

**Element**	**Aspect #1**	**Aspect #2**	**Aspect #3**
Biology and culture	Identifying specifics of biology and culture	Going beyond assumptions	Combining components
Say-do-process	What people say: Language, cognition, symbols	What people do: Movement, senses, actions and reactions	What people process: How outcomes/variation come to be
Sizing culture	Granularity of cultural lens: Macro, meso, micro, mental	Specific approach: symbolic, political economy, ecological	Identifying specific effects of culture
Cultural variation	Beyond WEIRD	Level of sharedness	Production of difference
Individuals and interactions	Individual: Biology, Psychology, Self, Social Self	Interaction: Embodiment, Practices, Context, Social Structures	Culture: How interactions hack brain processes
Interactive elements	Cultural practices that structure interactions	Materiality and patterning of interactive elements	How elements engage specific biological systems
Conceptual triangulation	Anthropology-Neuroscience-Psychology	Field-Lab-Clinic	Mind-Body-Culture
Critical complementarity	Draw on critical neuroscience, philosophy of science, cultural anthropology for critical analysis	Use complementary fields, such as cultural neuroscience and psychological anthropology	Complement strengths, Minimize analytical weaknesses
Methodological triangulation	Ethnographic methods, participants' perspectives, real-world empirical testing	Experimental, quantitative, biological, qualitative methods for assessing process	Include macro framing–culture, history, evolution–for interpretation of data

*These nine elements outline how to approach doing neuroanthropology. Researchers can work through the entire set in developing and executing their research. That said, different projects are likely to emphasize different elements as most relevant to their research problem. Specific elements can also be utilized for other types of research. Each element is condensed into three aspects that represent how to operationalize it for research. For example, cultural variation first implies getting beyond the individual-based and convenience-sample aspects of a lot of research (e.g., available university students), and then looks at shared aspects of variation and whether there are systematic differences within any particular aspect of shared variation*.

For Collura, considering cultural variation also made a difference. The research recognized the United States as unusual, as weird, in how it handles veteran transitions. Becoming a veteran is not a natural thing, but something mediated through culture, and thus related to the production of shared variation. A crucial insight came with recognizing that transitioning effectively was about finding new ways of generating shared variation. BJJ fostered exactly that. Finally, as Figure 2 illustrates, Collura pushed the say-do of normal ethnographic research into the say-do-process. The “mental + physical and social” model of veterans became a way to examine the processes involved in how BJJ worked to foster the production of shared variation.

**Figure 3:**
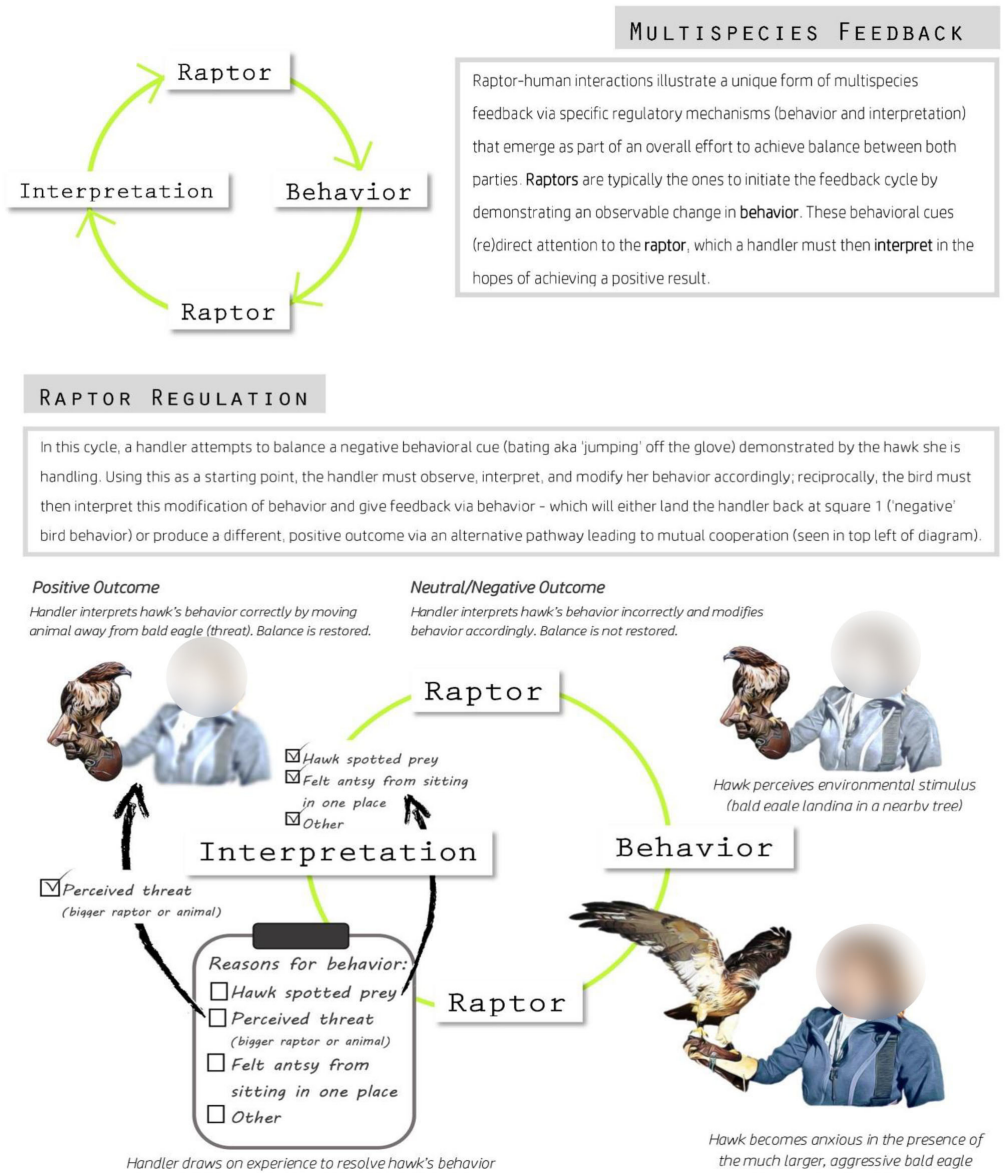
Multispecies feedback and raptor regulation.

The study of Hoyt first represents sizing culture. The work with raptors situated itself within an ecological approach, which fit well the animal-human interactions taking place at both institutional and individual levels, and how raptors can have agentive characteristics. Drawing on critical complementarity, the research navigated between insights coming from Animal Assisted Interventions research, interspecies anthropology, and the psychology of regulation. The critical part helped overcome assumptions about “lower animals” (like birds) that existed in some of the literature, as well as how animals can be anthromorphized. The complementarity part also helped connect AAI with psychology and anthropology. From there, the research identified specific interactive elements between the handler and the raptor that shaped mutual regulation, as shown in Figure 3. Specific behaviors and interpretations on both sides shaped both positive and negative outcomes.

**Figure 4:**
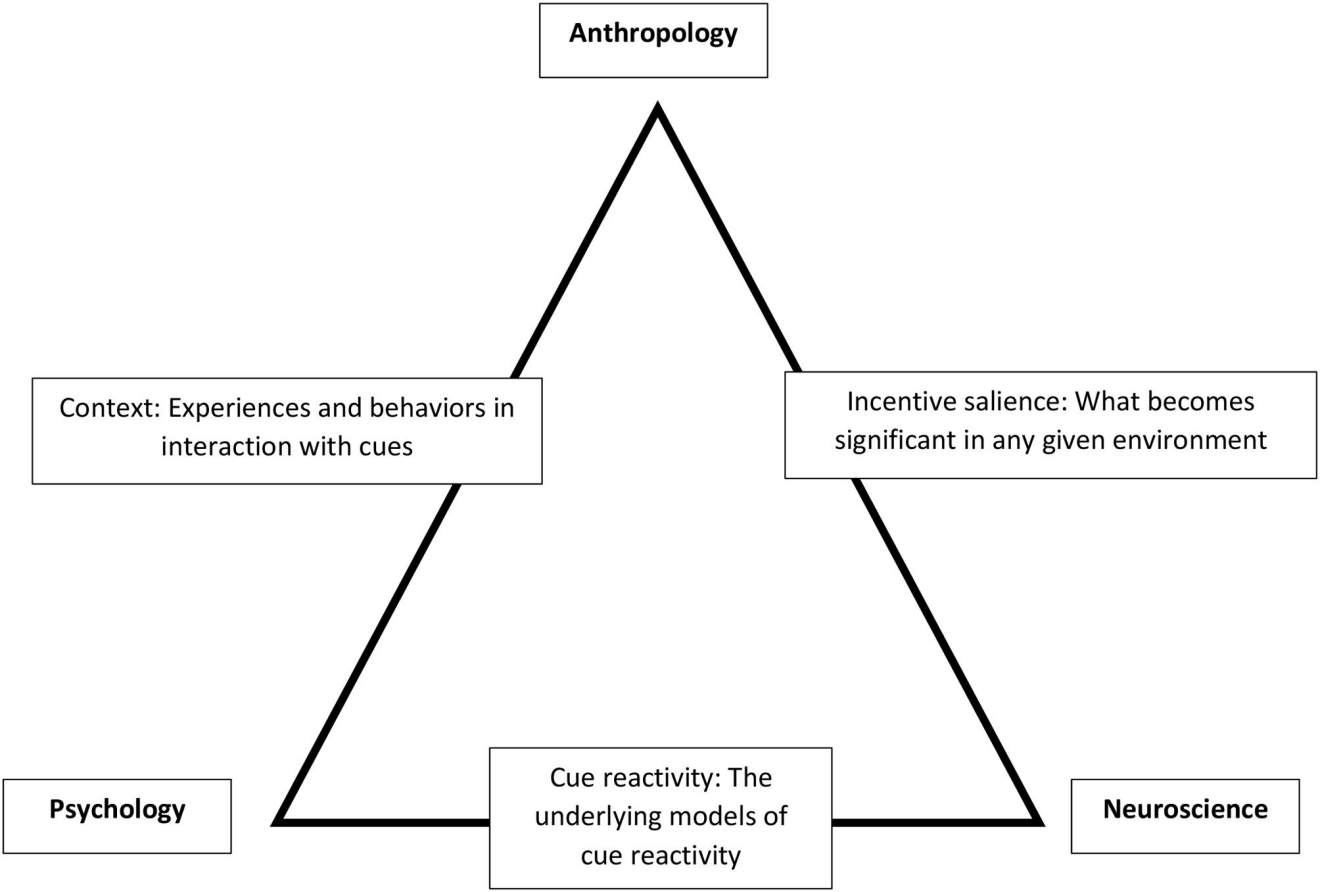
Theoretical triangulation for cue reactivity research. This figure demonstrates how to use theoretical triangulation to help develop aneuroanthropological approach to cue reactivity. Psychology and anthropology both look at human experience and behavior. For example, psychology lends insights into how humans interact specifically with cues while anthropology brings an understanding of how contexts can structure both cues and behavior. Psychology and neuroscience inform models of cue reactivity, including the psychophysiological basis of reactions to cues and cognitive models on the interpretation of cues. Neuroscience and anthropology overlap in the theoretical understanding of incentive salience, specifically understanding the relations between the external environment and the internal human environment.

The developing work by Casper drew on conceptual triangulation (Figure 4). Cue reactivity is a well-established approach in laboratory research but has not had as much impact on clinical practice; field-based work can address some of the shortcomings of laboratory work while also using real-world experiences to increase clinical relevance. Accomplishing that means drawing on anthropology as a crucial way to understand how cues work in real-world contexts.

Rather than relying on an exclusively cultural approach, however, this research considers how individuals and interactions in specific contexts can help tease apart how cues work and what types of interactions can drive reactivity. This framework moves beyond seeing “cue reactivity” as a thing in itself—a psychobiological phenomenon reducible based on learning theory and the pharmacological power of drugs—to examining how learning happens in specific contexts, how cues might get concentrated in specific arenas, and how interpretations can form part of why people react more or less.

To get at how reactivity adheres to individuals and learning and contexts requires using multiple methods together. Here, methodological triangulation provides a nested approach to address ideas about cue reactivity, to gather data that can relate to clinical relevance, and have data from multiple sources to aim to tease apart some of the broad conceptual framings that need to be tested against what actually happens with cues and reactivity in the field.

As this review of the three projects shows, successful research within neuroanthropology does not have to use the overall framework to get the job done. The nine features represent a set of guidelines and considerations that help promote successful research; however, specific emphases can be developed that are most related to the research problem and to the specific stage of the research. Indeed, for people interested in culture, mind, and the brain, specific elements will likely be more useful than others. Sizing culture might facilitate how to bring cultural insights to bear on what are often seen as individual problems, say-do process might help anthropologists develop new ways to view their ethnographic research, and critical complementarity might be useful for interdisciplinary research more generally.

For cultural science more broadly, we believe that by using this type of approach we can start to figure out the building blocks out of which patterns of variation are built both within and across societies. Neuroanthropology aims to go from a local to a comparative level and, from there, a comparative to a generalizable level. Good comparative work builds on good local work; good generalizations build from comparisons that accurately assess both the pattern of variation and a range of potential causes for that pattern. For many human problems, these patterns of variation emerge in the interaction of individuals with specific features of local culture and larger political economies and ecologies. Neuroanthropology offers an approach to understanding these types of human problems.

## Author Contributions

DL wrote the main sections of the manuscript. BC, KH, and GC wrote their corresponding sections. All authors contributed to the article and approved the submitted version.

## Conflict of Interest

The authors declare that the research was conducted in the absence of any commercial or financial relationships that could be construed as a potential conflict of interest.

## Publisher's Note

All claims expressed in this article are solely those of the authors and do not necessarily represent those of their affiliated organizations, or those of the publisher, the editors and the reviewers. Any product that may be evaluated in this article, or claim that may be made by its manufacturer, is not guaranteed or endorsed by the publisher.
